# One-step Solution Processing of Ag, Au and Pd@MXene Hybrids for SERS

**DOI:** 10.1038/srep32049

**Published:** 2016-08-25

**Authors:** Elumalai Satheeshkumar, Taron Makaryan, Armen Melikyan, Hayk Minassian, Yury Gogotsi, Masahiro Yoshimura

**Affiliations:** 1 Promotion Center for Global Materials Research (PCGMR), Department of Material Science and Engineering, National Cheng Kung University, Tainan, Taiwan R.O.C.; 2 Department of Materials Science and Engineering, and A.J. Drexel Nanomaterials Institute, Drexel University, Philadelphia, PA 19104, USA; 3 Russian-Armenian (Slavonic) State University, 0051, Yerevan, Armenia; 4 A. Alikhanian National Science Laboratory, 0036, Yerevan, Armenia

## Abstract

We report on one-step hybridization of silver, gold and palladium nanoparticles from solution onto exfoliated two-dimensional (2D) Ti_3_C_2_ titanium carbide (MXene) nanosheets. The produced hybrid materials can be used as substrates for surface-enhanced Raman spectroscopy (SERS). An approximate analytical approach is also developed for the calculation of the surface plasmon resonance (SPR) frequency of nanoparticles immersed in a medium, near the interface of two dielectric media with different dielectric constants. We obtained a good match with the experimental data for SPR wavelengths, 440 nm and 558 nm, respectively for silver and gold nanoparticles. In the case of palladium, our calculated SPR wavelength for the planar geometry was 160 nm, demonstrating that non-spherical palladium nanoparticles coupled with 2D MXene yield a broad, significanlty red-shifted SPR band with a peak at 230 nm. We propose a possible mechanism of the plasmonic hybridization of nanoparticles with MXene. The as-prepared noble metal nanoparticles on MXene show a highly sensitive SERS detection of methylene blue (MB) with calculated enhancement factors on the order of 10^5^. These findings open a pathway for extending visible-range SERS applications of novel 2D hybrid materials in sensors, catalysis, and biomedical applications.

Owing to their physicochemical properties, 2D materials such as graphene, layered metal oxides, sulfides, and nitrides have attracted much attention in SERS^1^. Graphene has recently been studied the most due to its unique 2D structure, superior mechanical strength, and attractive electronic and optical properties^2^. The surface plasmon resonances (SPRs) of graphene, however, are found to be in the infrared and THz range, limiting its electromagnetically modified (EM) enhancement in the visible range SERS^3^. Nevertheless, attempting to get compensation from chemically modified (CM) enhancement, graphene has been hybridized by *ex situ* and *in situ* methods with various nanomaterials including polymers, metal oxides and metal nanoparticles (NPs). For this, numerous hydrothermal, wet-chemical, electrochemical deposition, and photoreduction methods have been applied^4^,^5^,^6^,^7^,^8^,^9^. This can be useful not only in SERS, but also in biomedical, imaging, and sensing (even at a single-molecule level) applications.

In order to benefit from both EM and CM enhancements in the visible SERS applications, new 2D material hybrids are necessary. Some members of the MXene family, recently discovered early transition metal 2D carbides/nitrides^10^,^11^, are predicted to possess SPRs from infrared to ultraviolet (UV) range. MXenes are composed of layered P6_3_/*mmc* symmetry hexagonal M_n+1_X_n_T_x_ structures, where M is a transition metal or a combination of such, X is either C and/or N, T_x_ stands for the functional terminations (such as -O, -OH and -F), and n = 1, 2 or 3^12^. The plasmonic properties of MXenes are therefore dependent on the layers’ size, number and stacking order, as well as functional terminations^13^,^14^,^15^. To date, layered MXene nanosheets have been proposed for many applications including super capacitors, batteries, hydrogen storage and hybrid devices^16^,^17^. Particularly, exfoliated 2D hybrids with nanoparticles and polymers can be used as building blocks for fabricating novel functional hybrid materials^18^,^19^. Among them, the most notable is a MXene-Cu_2_O composite for catalytic applications, as well as a MXene-poly-vinyl alcohol (PVA) composite film, and a sandwich-like structure of MXene/carbon nanotube (CNT) paper for application as highly-capacitive battery anodes for energy storage^20^,^21^,^22^. The tunability of their chemical and physical properties renders MXenes very promising for hybridization with nanomaterials. Nevertheless, no MXene-based plasmonic nanostructures of any kind have been reported so far. On the other hand, hybridizing NPs has exhibited advantageous properties for SERS in the past decade due to their high surface to volume ratio, surface control, and excellent plasmonic properties^23^,^24^,^25^. The single delocalized *d*-electrons as well as surface roughness of NPs can selectively enhance electromagnetic radiation at specific excitation wavelengths. The enhancement factor can be further tuned by NP surface functionalization and grouping^9^,^26^. There have been numerous studies on colloidal solutions of NPs hybridized by graphene, 2D metal sulfides, and others^27^. Yoshimura *et al*. developed a soft processing (or soft solution processing) method with the aim to fabricate materials of desired shape, size, composition, and structure in solutions within a shorter time frame^28^,^29^,^30^,^31^. The method has already succeeded in producing large-scale functionalized 2D carbon-based materials for electrochemical and catalytic applications^5^,^32^. In this work, we extend this approach to achieve a one-step environmentally-benign hybridization of silver, gold and palladium NPs (Ag, Au and Pd) with Ti_3_C_2_T_x_ MXene in an aqueous solution, without an external reducing agent or surfactant, as illustrated in Fig. 1. We suggest a NP growth model and explain the plasmonic hybridization of NPs with MXene. We demonstrate the SERS applicability of the as-synthesized materials on methylene-blue (MB), a well-known dye used as a probe molecule.

## Results

**Figure 2:**
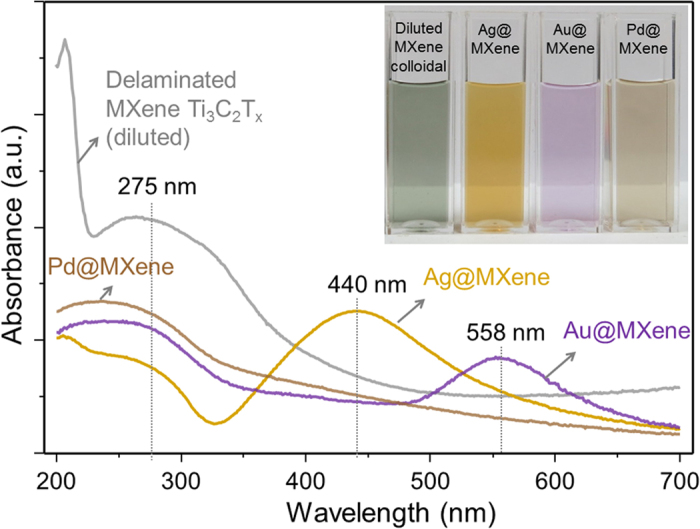
UV-Vis spectroscopy analysis on the delaminated Ti_3_C_2_T_x_ nanosheets (MXene) and the colloidal suspension of MXene-metal hybrid materials. A photograph of solutions of the diluted MXene colloid, Ag, Au and Pd NPs@MXene colloids is presented in the inset (left to right, correspondingly).

Figure 2 shows ultra-violet and visible (UV-Vis) spectroscopy results on delaminated Ti_3_C_2_T_x_ MXene (0.04 mg/mL concentration) and colloidal MXene-NP hybrid suspensions derived from it. It can be seen from the photographs (inset of Fig. 2) that the Ag, Au and Pd@MXene hybrid colloidal solutions are well dispersed in water. The broad UV absorption spectrum of the delaminated MXene in dilute aqueous medium exhibits peaks at 225 and 275 nm in Fig. 2. This is expected because the surface of MXene was functionalized with different groups after etching out the A-element from its precursor ternary transition metal carbide (so called MAX phase) during synthesis, and the flakes were delaminated by dimethyl sulfoxide (DMSO). The MXene colloid also exhibits high absorption in the UV region within the range from 225 to 325 nm. This absorption may correspond to the band-gap energy of the oxidized MXene, which was also predicted by theoretical calculations^16^,^17^. The SPR bands of Ag and Au@MXene suspensions appear in the visible region, at 440 and 558 nm, respectively. This indicates the presence of NPs (Ag and Au, respectively) in the colloidal solution. Also, it shows that an advantageous EM enhancement can be expected from these NP@MXene hybrid structures. The SPR band for Pd NPs is usually observed in ultraviolet region^33^. In the case of the Pd@MXene, a single UV peak is observed at 230 nm, which could be considered a red-shifted MXene peak due to the coupling with the deposited Pd NPs, as shown in the discussions section.

**Figure 3:**
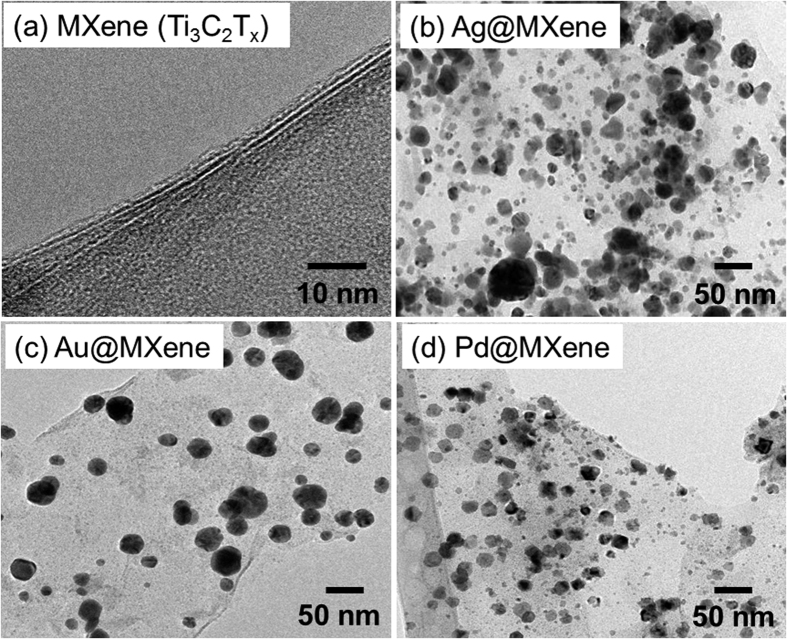
(**a**) High resolution TEM image of MXene nanosheets and low resolution TEM images of (**b**) Ag@MXene, (**c**) Au@MXene and (**d**) Pd@MXene hybrid nanosheets.

To visualize the morphology of the as-synthesized NPs on MXene, we performed transmission electron microscopy (TEM) analysis. Moreover, to highlight the constitution of these NPs, we simultaneously performed energy dispersive X-ray (EDX) mapping and spectroscopy (EDS) on the same areas as analyzed by TEM. The cross-section of layered MXene structure (Fig. 3a) and the distributed NPs (Ag, Au and Pd) on MXene flakes are shown in the TEM micrographs in Fig. 3b–d, respectively. The bare MXene sample consists of single- and few-layer MXene flakes (Fig. 3a) with lateral sizes around 2–3 μm (Supplementary Fig. S1a). The corresponding high-resolution TEM images of MXene indicate thickness from one to few nanometers (Supplementary Fig. S1b). The EDS spectrum and EDX mapping reveal that the delaminated MXene consists of only Ti and C with no Al (Supplementary Fig. S1), suggesting successful etching. From the high resolution TEM micrographs (Supplementary Figs S2–S4), it is clear that the NPs were directly attached to the MXene. The size and shape of the NPs vary for different metals synthesized in this study. The Ag and Au NPs are rounded with sizes varying from 10–70 and 40–50 nm, respectively. The size of Ag NPs on MXene is harder to control because the highly-reactive Ag^+^ ions are likely to undergo rapid reduction under the ultrasonication even when using a low precursor concentration (0.1 mM AgNO_3_ solution). Nevertheless, under the same experimental conditions, a more homogenous size distribution of Au NPs (40–50 nm in average) was obtained, suggesting a slower reduction of Au^3+^ ions. Interestingly, in the Pd@MXene case, high resolution TEM imaging reveals sheet-like planar particles on the surface of MXene flakes (Supplementary Fig. S4), suggesting that the reduction step was different from that of the Ag and Au. The distribution and elemental EDX mapping analysis for each MXene-NP hybrid confirmed the presence of the synthesized metal nanoparticles on MXene (Supplementary Figs S2–S4).

**Figure 4:**
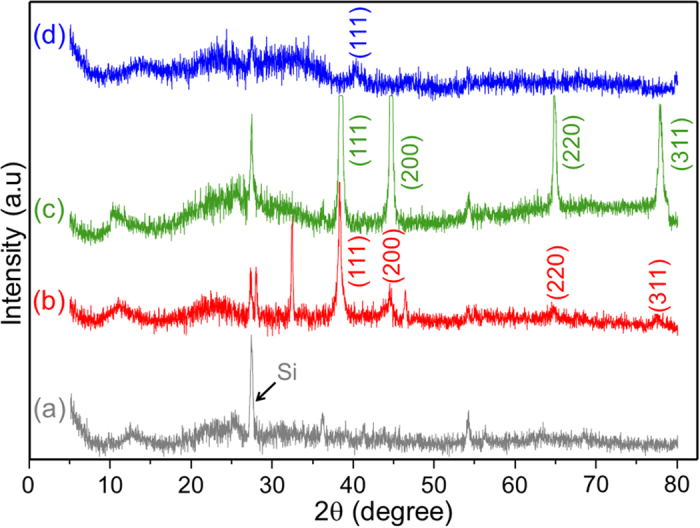
The powder X-ray diffraction patterns of (**a**) delaminated MXene nanosheets, and after hybridization with (**b**) Ag, (**c**) Au and (**d**) Pd nanoparticles.

To assess the structure of the metal NPs on the MXene, we performed X-ray diffraction (XRD) analysis before (Fig. 4a) and after hybridization of MXene (Fig. 4b–d) with Ag, Au, and Pd, as shown in Fig. 4. To compare with the same signal to noise ratios, the XRD patterns of the NP@MXene hybrids were normalized with respect to the sharp Si substrate peak at 27° (2θ). The XRD patterns of the delaminated MXene have very weak peaks comparable to those previously reported^12^. The peak of MXene at 13° (2θ) shifts to 11° and 9° in the cases of Ag@ and Au@Mxene hybrids, respectively. The disappearance of the non-basal peak at ~60° (2θ) suggests the full delamination of MXene and no crystallographic stacking of MXene sheets^12^. We also found intense peaks corresponding to the (111), (200), (220) and (311) planes of the Au and Ag NPs, which further confirm the successful hybridization. The right-shifted (111) peak of Pd@MXene indicates the different hybridization mechanism of the planar NPs. The nm-range thickness of the planar NPs hinders the detection of reflected light from planes other than the (111). These observations are consistent with the shape analysis performed by TEM, in Fig. 3.

**Figure 5:**
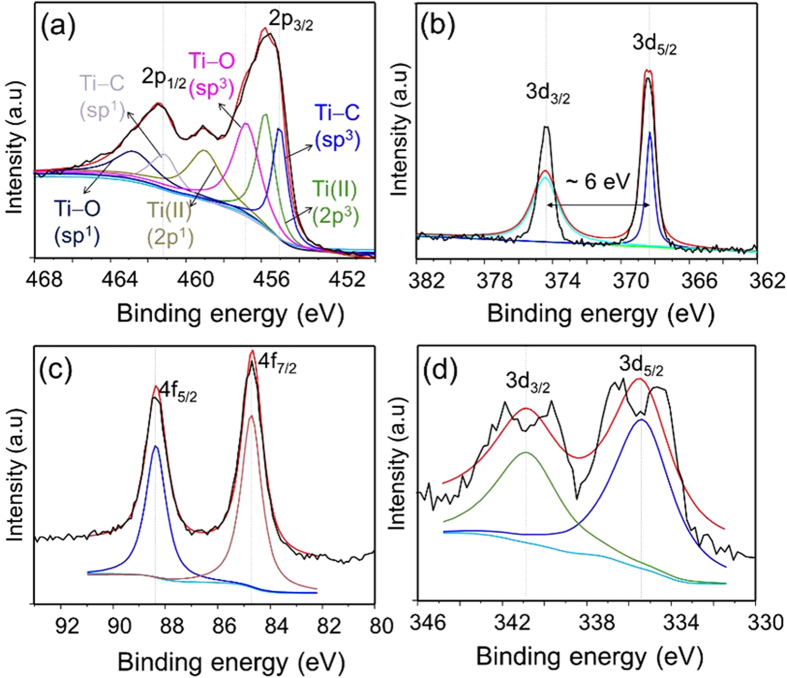
Deconvolution of high-resolution XPS spectra for the elements in (**a**) delaminated MXene, and (**b**) 3d core level spectrum for Ag@MXene, (**c**) 4f core level spectrum for Au@MXene and (**d**) 3d core level spectrum for Pd@MXene.

The surface chemical bonding of the MXene and MXene-hybrids were examined by X-ray photoelectron spectroscopy (XPS). The individual core level high-resolution XPS deconvolution spectra have been background corrected using the Shirley algorithm prior to curve resolution, presented in Fig. 5.

**Figure 6:**
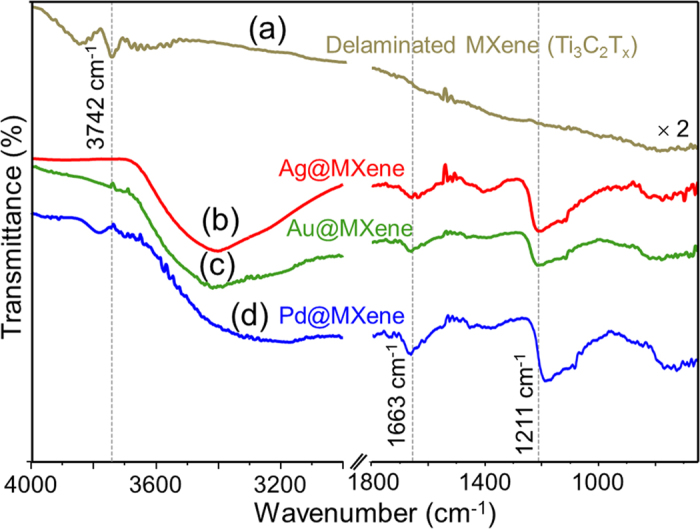
ATR-FTIR spectroscopy analysis of delaminated MXene nanosheets (Ti_3_C_2_T_x_) and (**b**) Ag@, (**c**) Au@ and (**d**) Pd@MXene hybrids.

As seen from the high-resolution XPS spectra of Ti 2p, the O and C contribution mainly comes from the TiO_x_ and carbon of TiC, respectively. Moreover, the peaks at 454.9 eV (sp^3^) and 461.2 eV (sp^1^) represent a contribution from Ti–C bonds, and the peaks at 456.6 eV and 462.7 eV correspond to Ti-O bonds. Figure 5b shows the 3d Ag core level spectrum with doublet peaks of Ag 3d_5/2_ and 3d_3/2_ of the two chemically distinguished spin-orbit pairs observed at 368.4 eV and 374.4 eV, respectively. The low binding energy component (at 368.4 eV) is a characteristic peak for electron emission from the Ag nanocore. The difference in binding energy (~6 eV) of this doublet clearly shows evidence of the Ag atoms present in the drop-coated film. Likewise, the deconvolution core level spectrum of the Au@MXene hybrid (Fig. 5c) shows peaks at 84.7 eV and 88.4 eV in which the difference in binding energy (3.7 eV) indicates a reduced form of Au^0^, further confirming the presence of Au nanoparticles in the sample. An analogous conclusion about Pd nanoparticles can be drawn from the Pd@MXene Pd core levels at 335.4 eV and 340.8 eV, and their difference (Fig. 5d). The back-scattered light from planar Pd nanoparticles is, however, screened by the MXene flakes, causing the low signal-to-noise ratio. We also inspected the XPS deconvolution core level spectra of Ti 2p, C 1s and O 1s of MXene and MXene-hybrids. A survey spectrum of MXene revealed the presence of Ti, C and O atoms (Supplementary Fig. S5). The C 1s and O 1s spectra of MXene suggest that after the delamination process, the MXene surface was predominantly functionalized with OH groups, the contributions from graphitized carbon, and carbides (Supplementary Figs S7 and S8, respectively). The functional groups of the delaminated MXene and MXene-hybrids were further analyzed by Fourier-transmission infrared (FTIR) spectroscopy in the attenuated total reflection (ATR) mode, as displayed in Fig. 6. The IR spectrum of the delaminated MXene has a vibrational mode at 3742 cm^−1^, which, due to the absence of atmospheric moisture (the samples were blow-dried in N_2_ flow prior to the measurements), is assigned to the -OH functional group out-of-plane vibrations of MXene. This is verified by first-principles calculations of Ti_3_C_2_(OH)_2_ in the Ti_3_C_2_ block^34^. The IR absorption spectra of NP@MXene hybrids have three broad peaks corresponding to vibrational bands at 3400–3800, 1661, and 1211 cm^−1^. These are assigned to the OH/H_2_O adsorbed on the NPs surface, since these bands were not observed in the case of delaminated MXene flakes.

## Discussion

The hybridization of exfoliated 2D nanosheets is potent not only in tailoring the physicochemical properties of hybridized species, but also in showing catalytic activity in concert with coupling between the 2D materials and NPs. Au NPs were previously deposited on delaminated MXene flakes with sodium borohydrate (NaBH_4_) as a reducing agent^35^. Moreover, it was observed that a homogeneous size distribution was achieved without a reducing agent by direct addition of Au salt in dark conditions or by photocatalytic assistance of UV irradiation^35^. Metal NPs (Pt) were also deposited directly on MXene flakes^36^. Herein, we propose a general *in situ* reduction method of noble metal NPs on MXene flakes by direct addition of metal salts in ambient air. Firstly, the effect of sonication on the hybridization of MXene flakes was systematically tested by performing treatments both with and without ultrasonication. The *ex situ* TEM analysis revealed that a homogeneous deposition of NPs (on individual MXene flakes) and subsequently, highly dispersed MXene-hybrids (case of Au) were accomplished by ultrasonication. In the case when ultrasonication was not used, non-uniformly deposited NPs on stacked MXene flakes (>4 nanosheets) were observed (Supplementary Fig. S9). Thus, we conclude that the ultrasonic mixing helped to obtain a more uniform NPs distribution on finer dispersed MXene nanosheets.

**Figure 7:**
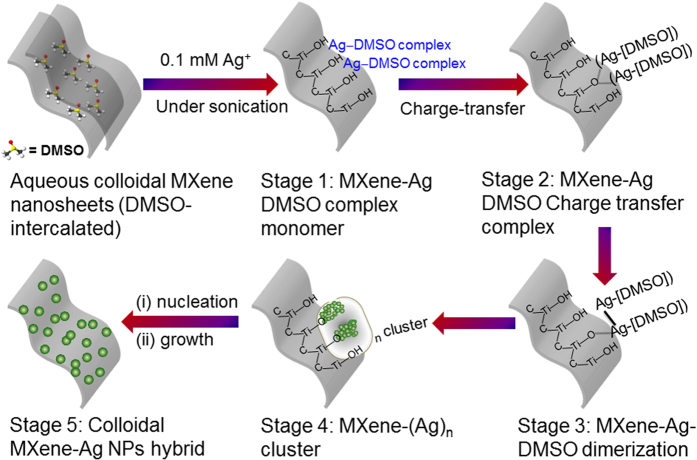
The graphical representation of the reduction mechanism of Ag nanoparticles on Ti_3_C_2_T_x_ MXene nanosheets (Ag@Ti_3_C_2_T_x_) via sonochemical approach by the *in situ* one-step solution processing synthesis.

Further, considering all the performed analyses, we propose a reaction mechanism for the *in situ* formation of Ag@MXene hybrids by the developed approach, Fig. 7, involving Ag-DMSO complexes. We note that based on preliminary results of^35^ another salt-reduction process without the DMSO complexes could possibly occur on the MXene flakes, either by or without photocatalytic activation. Nevertheless, we hypothesize hereby a mechanism involving DMSO intercalants attached to the few nm-thick delaminated MXene flakes^12^ serving as reduction/nucleation sites for the NPs^37^. As metal ions (0.1 mM Ag^+^) are added into the MXene colloidal solution under sonication, the formation of [Ag^+^-DMSO] complex monomers occurs in the MXene solution (stage 1). The [Ag^+^-DMSO] complexes cause an immediate color change by forming Ag^+^-DMSO (monomer) strong surface bonds with the -OH^−^ functional groups of MXene. Thereafter, a fast electron transfer causes the formation of Ag^+^-[DMSO] from oxygen’s lone-pair electron ([ÖS-(CH_3_)_2_]) (stage 2). In the next step (stage 3), the charge transfer between Ag_1_-MXene complexes initializes the MXene-Ag DMSO dimerization. Since the bond with the -OH functional group is stronger, this MXene-Ag dimeric complexes then undergo a further reduction and form stable (Ag^0^)_n_ nano clusters (stage 4). This cluster formation leads to further nucleation and subsequent growth to form stable Ag NPs on the MXene surface (stage 5). Eventually, this MXene localized surface-reduction mechanism yields plasmonic 2D substrates decorated by NPs that are highly dispersed in aqueous solution. The coverage of the NP can be further tuned by chemical manipulation of the –OH functional groups on the MXene substrate.

Next, to understand the nature of the noble metal NPs plasmon peaks observed by the UV-vis spectroscopy, as shown in Fig. 2, we calculated in the limits of quasistatic approximation the SPR frequency of an Ag or Au sphere placed near the interface of two dielectric media with dielectric constants denoted as ε_a_ and ε_b_. The case of Pd is discussed separately. We apply the imaging method^38^ to reduce the problem of the NP immersed in ε_a_ medium near the MXene (ε_b_) sheet to a problem of two NPs in a homogeneous medium. For solving this reduced problem, we involve the approximate analytical approach developed for two spheres^39^. We consider a NP of a radius (R) in the range of 5 to 35 nm where the developed quasistatic approximation is valid. Considering the charge distribution coefficient of the image NP, 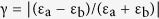, (Supplementary information), we obtain the following relation:


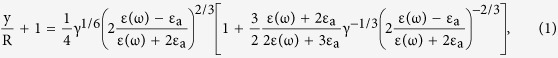


where y is the minimal distance between NP and MXene, ε(ω) is the dielectric function of the NP depending on frequency ω. Eventually, inserting experimental values of dielectric constants^15^,^40^ in equation (1), we obtain a good match with the experimental data for SPR wavelengths, 440 nm and 558 nm, respectively for Ag and Au, in the case of a reasonable value of y/R ≈ 0.015. Our calculations (Supplementary information) also show that the SPR linewidths of Fig. 2 are conditioned mainly by radiation damping in the case of Ag, and by interband transitions (with a ~30% contribution from radiation damping) in the case of Au@MXene hybrids.

As seen from Fig. 3, the case of Pd structures deposited on MXene flakes drastically differs from that of the Ag and Au NPs. According to the TEM analysis results, we model these Pd nanostructures by highly oblate spheroids (disc-shaped particles). By using Pd optical constants^41^ and our calculated value of the effective refractive index (Supplementary information), we obtain a SPR peak in the UV range at 160 nm. The more sophisticated planar NP geometry and coupling can indeed yield a more red-shifted SPR value, evident in Fig. 2. Nevertheless, when we model Pd NPs by spheres of the same size as those of the Ag and Au NPs, we obtain an SPR of 375 nm, which is in accordance with the literature^42^. The absence of such a peak in Fig. 2 for Pd@MXene, however, confirms the non-spherical shape of the Pd NPs.

**Figure 8:**
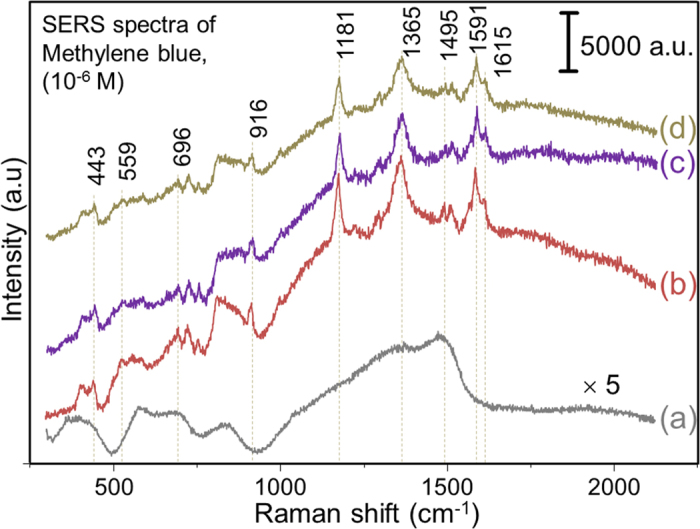
(**a**) Raman spectrum of Ti_3_C_2_T_x_ after soaking in MB dispersed in ethanol and subsequent drying. SERS spectra of MB with (**b**) Ag@, (**c**) Au@ and (**d**) Pd@MXene.

Finally, to demonstrate the SERS sensitivity of the NP@MXene hybrids against MB probe molecules, we performed Raman measurements on MXene before (Fig. 8a) and after NP hybridization (Fig. 8b–d) by soaking the glass-deposited samples in a 10^−6^ M ethanol solution of MB and subsequently drying it. In order to improve the signal to noise ratio of the detected Raman signal, we use a hybridization of NPs with increased particle size and amount by adding a 5-fold raised (0.5 mM) concentration of each precursor metal salt during the hybridization process. This could result in a fast reduction of the precursor, causing non-uniform and larger size NP coating (possibly aggregated) on the MXene surface. The self-assembled monolayers of MB were formed on MXene and MXene hybrids (Ag@, Au@ and Pd@MXene) deposited onto a glass substrate. Characteristic Raman peaks of partially-oxidized Ti_3_C_2_T_x_^10^,^16^ can be seen in Fig. 8a. The oxidation could have occurred due to reaction with ethanol or MB, or from the high laser power (35 mW). Nevertheless, no Raman spectral features of MB can be seen in Fig. 8a even though MB molecules were adsorbed on MXene. This clearly comes from the absence of the NPs on the MXene surface, which are necessary for enhancing the Raman signal of MB. The Raman features of MXene are no more evident in the NP@MXene hybrids, possibly due to the much larger Raman scattering cross-section of the covering MB molecules. The characteristic peaks of MB around 443 and 1615 cm^−1^ which have been assigned to C–N–C skeletal bending and C–C stretching, respectively, are evident in the SERS spectra for the three NP hybrids (with varying intensities), indicating that the molecules were adsorbed onto the substrates^43^. The SERS characteristic bands for δ (C-S-C) and υ(C–S) at 559 cm^−1^ and 1181 cm^−1^, respectively, are observed suggesting that the chemisorbed MB molecules were attached to the MXene-hybrids surface via sulphur–metal bond, enriching C-S bond intensities. In addition, several bands, particularly for Ag@MXene hybrid, such as δ(C–H) at 696 cm^−1^, υ_sym_(C-N) at 1365 cm^−1^, υ_asym_(C-N) at 1495 cm^−1^, aromatic υ_asym_(C-C) at 1516 cm^−1^ and υ_sym_(C-C) at 1591 cm^−1^ can be identified. These MB bands appear in the case of Au and Pd@MXene hybrids with slight shifts, indicating the CM nature of these SERS effects, presented in Fig. 8c,d. The produced 2D MXene-hybrid substrates are hence expected to exhibit high SERS enhancement. As a reference, 1% of liquid MB (in ethanol) was used to acquire a Raman spectrum for the calculations of the enhancement factors of 2D MXene-hybrids samples (Supplementary Fig. S10). For instance, the calculated enhancement factors for Ag@, Au@ and Pd@MXene reach 1.50 × 10^5^, 1.17 × 10^5^ and 9.61 × 10^4^, respectively. This suggests possible SERS applications of the proposed material. The detailed SERS enhancement (both, CT and EM) mechanism of adsorbed probe molecules on single layer Ti_3_C_2_T_x_-hybrid (in the case of Au@Ti_3_C_2_T_x_) is under investigation.

In conclusion, herein we report a strategy for synthesis of noble metal nanoparticle-MXene hybrids by one-step soft solution processing. Through TEM and simultaneous EDX mapping analysis, we show that a uniform coating of Ag, Au and Pd NPs on MXene nanosheets was accomplished. By combining various spectro-chemical characterizations, we propose a mechanism of hybridization of MXene nanosheets for Ag@MXene. The plasmonic nature of these novel 2D hybrid nanostructured substrates was demonstrated by surface enhanced Raman scattering (SERS). Eventually, under the preparation conditions optimized in this work, the produced Ag@MXene, Au@MXene and Pd@MXene substrates demonstrated high sensitivity to methylene blue molecules with calculated enhancement factors of 1.5 × 10^5^, 1.17 × 10^5^ and 9.61 × 10^4^, respectively. The advantage of this method is the *in situ* controlled synthesis approach, as well as the EM and CM combined nature, favorable for the SERS enhancement. Moreover, the simplicity of the solution-based approach and the absence of reducing agents and expensive instrumentation allow exploration of other MXene-NP hybrids and for applications including optical sensors (colorimetric detection), electrochemistry, catalysis and interface studies.

## Methods

### Synthesis of aqueous MXene nanosheets colloidal solution

The MXene powder of Ti_3_C_2_T_x_ was prepared by etching with a fluoride salt (LiF in HCl) at 35 °C for 24 h from Ti_3_AlC_2_ (MAX phase). The obtained MXene powder was used for the production of colloidal suspension of MXene nanosheets by delamination of powder and subsequent bath sonication (Bransonic M3800 Ultrasonic bath, 110 W and Frequency 40 kHz). For delamination, 0.3 g of MXene powder was mixed in 5 mL DMSO and then magnetically stirred at room temperature (RT) for 24 h. The DMSO-intercalated Ti_3_C_2_T_x_ suspension was centrifuged at 3,500 rpm for 5 min. After decantation of the supernatant of liquid DMSO, 150 mL of deionized water was added to the residue of DMSO-intercalated Ti_3_C_2_T_x_. This aqueous suspension of Ti_3_C_2_T_x_ was sonicated for 10 h and then centrifuged at 3,500 rpm for 1 h to obtain supernatant containing Ti_3_C_2_T_x_ nanosheets, referred to as MXene nanosheets colloidal solution.

### Preparation of MXene nanosheets-based plasmonic nanocomposites

The 200 μL of original Ti_3_C_2_T_x_ nanosheets colloidal solution was re-dispersed in 10 mL of distilled water under sonication. In this mixture, an aqueous AgNO_3_, HAuCl_4_ and PdCl_2_ salt solutions were added with a concentration of 0.1 mM (for Ag, Au, and Pd@MXene hybrid structures, respectively) and then sonicated for 10 min. The obtained colloidal solution of hybrid nanocomposites of Ag, Au, and Pd@Ti_3_C_2_T_x_ hybrids were centrifuged at 10,000 rpm for 5 min and re-dispersed in deionized water for further analysis. The colloids were stored at RT.

### Characterization

The Ti_3_C_2_T_x_ MXene, and Ag, Au, and Pd@Ti_3_C_2_T_x_ hybrid nanocomposites colloidal solutions were monitored by a double beam UV–vis spectrophotometer model U-3000 (Hitachi, Ltd., Tokyo, Japan). Fourier-transformed infrared (FT-IR) spectroscopy with ATR accessory (Vertex 70, Bruker, Germany) was used for recording spectra in a range of 400 to 4000 cm^−1^ for MXene and hybrid samples. Before the FTIR analysis, the MXene and MXene-hybrids samples were dried at room temperature with N_2_ gas to remove atmospheric moisture. TEM (HT7700, Hitachi, Tokyo, Japan) was used to study the morphology of Ti_3_C_2_T_x_ and hybrid Ag@, Au@ and Pd@Ti_3_C_2_T_x_ nanostructures. For that, the samples were prepared by drop-casting the re-dispersed colloidal solutions of Ti_3_C_2_T_x_, Ag, Au and Pd@Ti_3_C_2_T_x_ hybrids onto a carbon film-coated 300 square mesh copper grid. High resolution XPS measurements were carried out on a PHI Quantera SXM spectrometer (ULVAC Inc., Kanagawa, Japan), and PF4 software was used for the deconvolution of the narrow-scan XPS spectra. XPS was performed with a monochromatic Al Ka source (25 W, hυ = 1486.6 eV) and an energy resolution of 1 eV. The XRD patterns were recorded by a Bruker D2-phaser diffractometer (Karlsruhe, Germany) using Cu Kα radiation (*λ* = 1.5418 Å). For the XRD and XPS analysis, the samples were prepared by depositing re-dispersed MXene nanosheets and plasmonic hybrid nanocomposites onto a silicon substrate and drying them at 60 °C for 30 min.

### SERS measurement

MXene and MXene hybrids (Ag, Au and Pd@MXene) colloidal samples were deposited onto glass substrates by drop-casting method and dried in ambient air at 60 °C for 30 min. The self-assembled monolayers of methylene blue (MB, 10^−6^ M) were formed on Ag, Au, and Pd@MXene hybrids by soaking in ethanolic solution of MB for 60 min. The Raman spectra were collected using a Triax 320 system (Jobin-Yvon Inc., Longjumeau, France), which was equipped with a 35 mW, 632.8 nm He/Ne laser (JDS Uniphase Co., Milpitas, CA) and a liquid-nitrogen-cooled Ge CCD array detector (Jobin-Yvon Inc.) at a resolution of 0.06 nm. The spectral acquisition time was 5 s and 20 s for all the SERS and normal Raman measurements with 2 accumulations averaged. The wavenumber was calibrated using the Si peak at 520 cm^−1^ as a reference.

## Additional Information

**How to cite this article**: Satheeshkumar, E. *et al*. One-step Solution Processing of Ag, Au and Pd@MXene Hybrids for SERS. *Sci. Rep.* **6**, 32049; doi: 10.1038/srep32049 (2016).

## Supplementary Material

Supplementary Information

## Figures and Tables

**Figure 1:**
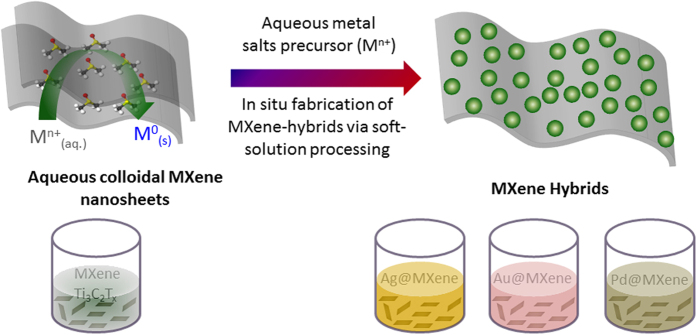
The graphical representation on *in situ* one-step solution processing synthesis Ag, Au and Pd@MXene (Ti_3_C_2_T_x_) hybrids by soft-solution processing via sonochemical approach.
