# Author Correction: One-step Solution Processing of Ag, Au and Pd@MXene Hybrids for SERS

**DOI:** 10.1038/s41598-020-72793-y

**Published:** 2020-09-25

**Authors:** Elumalai Satheeshkumar, Taron Makaryan, Armen Melikyan, Hayk Minassian, Yury Gogotsi, Masahiro Yoshimura

**Affiliations:** 1 Department of Material Science and Engineering, Promotion Center for Global Materials Research (PCGMR), National Cheng Kung University, Tainan, Taiwan, ROC; 2 Department of Materials Science and Engineering and A.J. Drexel Nanomaterials Institute, Drexel University, Philadelphia, PA 19104 USA; 3 Russian-Armenian (Slavonic) State University, 0051 Yerevan, Armenia; 4 A. Alikhanian National Science Laboratory, 0036 Yerevan, Armenia

Correction to: *Scientific Reports* 10.1038/srep32049, published online 25 August 2016

In this Article, Figure 4 contains an inappropriate base line correction and signal-to-noise (S/N) ratio duplication at low angle between the 5–10 degrees (2θ). The correct Figure 4 without any spectral processing appears as Figure 1.

**Figure 1:**
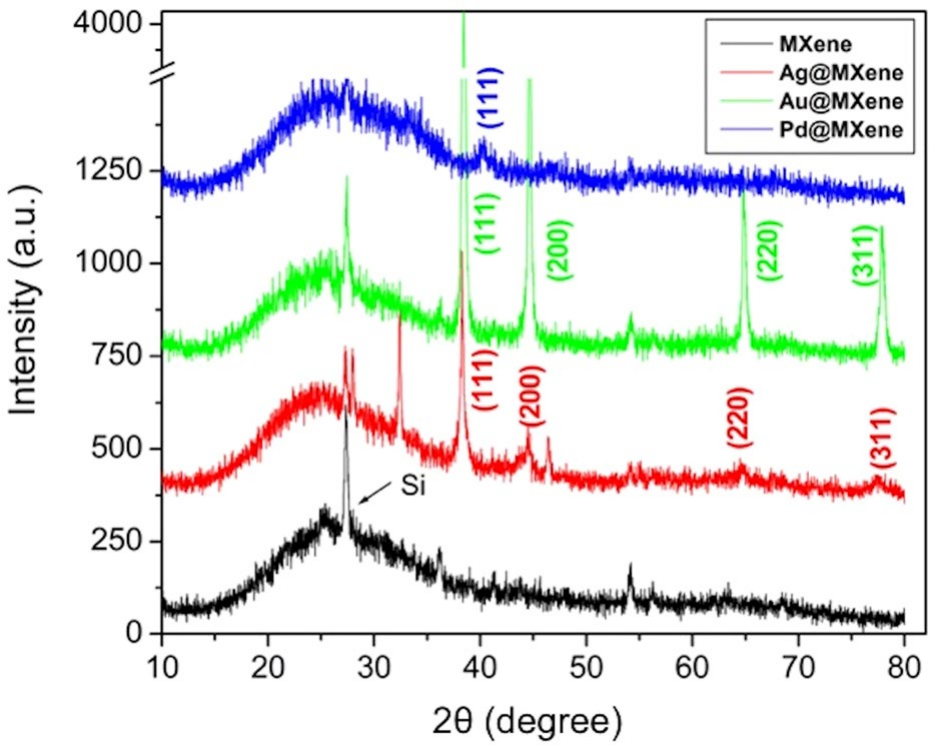
.

As a result, in the Results section,

“The XRD patterns of the delaminated MXene have very weak peaks comparable to those previously reported^12^. The peak of MXene at 13° (2θ) shifts to 11° and 9° in the cases of Ag@ and Au@Mxene hybrids, respectively.”

should read:

“The XRD patterns of the delaminated MXene have very weak peaks comparable to those previously reported^12^.”

