# Supplementary material for: One-step Solution Processing of Ag, Au and Pd@MXene Hybrids for SERS
Source: Sci Rep. 2016 Aug 25;6:32049. doi: 10.1038/srep32049 (PMC4997347; doi:10.1038/srep32049)
Supplement: Supplementary Information [file srep32049-s1.pdf]

Supplementary Information

One-step Solution Processing of Ag, Au and Pd@MXene Hybrids for SERS

Elumalai Satheeshkumar[1], Taron Makaryan[2], Armen Melikyan[3], Hayk Minassian[4], Yury Gogotsi[2*] & Masahiro Yoshimura[1*]

[1] Promotion Center for Global Materials Research (PCGMR), Department of Material Science and Engineering, National Cheng Kung University, Tainan, Taiwan R.O.C, E-mail: yoshimur@mail.ncku.edu.tw

[2] Department of Materials Science and Engineering, A.J. Drexel Nanomaterials Institute, Drexel University, Philadelphia, PA 19104, USA

[*]Email: gogotsi@drexel.edu

[3]Russian-Armenian (Slavonic) State University, 0051, Yerevan, Armenia

[4]A. Alikhanian National Science Laboratory, 0036, Yerevan, Armenia

---

*Prof. Masahiro Yoshimura E-mail: yoshimur@mail.ncku.edu.tw

Tel: +886-6-2757575 ext. 62013

*Prof. Yury Gogotsi, E-mail: gogotsi@drexel.edu

Tel 1-215-895-6446, Fax 1-215-895-1934

Calculation of the enhancement factor:

The enhancement factor (EF) is calculated using the equation,

$[I_{SERS}/C_{SERS}]/[I_{Normal}/C_{Bulk}]$ ……………………….(1)

where I_{SERS} and I_{Normal} were the intensity of the surface enhaced Raman scattering

(SERS) (at 1181 cm^{-1}) and normal Raman spectra of the probe molecule, respectively. C_{SERS} and C_{Bulk} are the concentration of probe molecule in the SERS and bulk samples, respectively.

Calculation of Electromagnetically modified EF of the Ag@MXene

Excitation wavelength = 632.8 nm

Power of laser = 35 mW

Laser diameter (cm) = 2*632.8 nm /(π*NA) = 0.052 cm

Laser area (cm^2) = 0.052 * 0.052 * 3.1415926 (πr^2)

= 0.008494866 cm^2

Sample holder diameter (cm) = 0.805 cm

Laser pass volume (cm^3) = Area * diameter

= 0.052 cm * 0.008494866 cm^2

= 0.006838367 cm^3

Number of methylene blue (MB, with 1 %)

Molecules in bulk = [(0.1/319.85/10)*(6.02*10^{23})]

= 1.88213E+19

M-bulk, Number of molecules in

Laser pass (cm^3) = [Laser pass volume * Number of MB molecules]

= 1.28707E+17

The number of molecules per unit area (Molecules per area)

= [Conc. of MB (5.00E-04 M)*Vol. of MB (5.00E-06, L)*6.02*10^{23}/ (0.75*1)]

= 2.00667E+15

M_{ads} - Laser through the sample molecules

(Number of molecules in laser passes) =[number of molecules per unit

area/laser area]

= [(2.00667E+15)/ (0.008494866)]

= 1.70464E+13

Normal Raman (1 % MB in ethanol, 20 sec)	= 1086 (a.u)
SERS (MB@Ag@MXene, 20 sec)	= 21629 (a.u)

**Enhancement Factor**	$=[I_{SERS}/C_{SERS}]/[I_{Normal}/C_{Bulk}]$
	=[(21629)/(1.70464E+13)/(1086)/ (1.28707E+17)]
	= 1.5038E+05 (± 0.108)

Similarly, the EFs for Au@MXene and Pd@MXene reach 1.17 E+05 and 9.61 E+04, respectively.

Calculation of the observed surface plasmon resonant (SPR) peak positions and widths in Ag@MXene and Au@MXene.

Interpretation of the SPR Peak positions: The relation between SPR frequency (ω_{SPR}) of two noble metal nanospheres and interparticle distance, x, is given (equation 45, [i]) as follows:

$$\frac{x}{2\sqrt{R_1R_2}} = \left\{\frac{1}{4R_1R_2}(4\alpha_1\alpha_2)^{\frac{1}{3}}\left[1+\frac{3}{2}R_2^5\frac{\beta_2}{\alpha_2}(4\alpha_1\alpha_2)^{-\frac{1}{3}}\right]\left[1+\frac{3}{2}R_1^5\frac{\beta_1}{\alpha_1}(4\alpha_1\alpha_2)^{-\frac{1}{3}}\right]\right\}^{\frac{1}{2}} - \frac{R_1+R_2}{2\sqrt{R_1R_2}} \qquad (2)$$

where R_1 and R_2 are the sphere radii, $\varepsilon_{1,2}(\omega)$ are the dielectric functions of the NPs, $\alpha_{1,2}$ are the dielectric polarizability of the metals and $\beta_{1,2} = (\varepsilon_{1,2}(\omega) - \varepsilon_a)/(2\varepsilon_{1,2}(\omega) + 3\varepsilon_a)$. If a metal NP residing in a ε_a dielectric

constant medium is placed near a separation interface with a ε_b medium, then its image will appear as a same-size NP at the same distance from the interface, and the charge distribution on it will differ from the original NP by the coefficient

$\gamma = \left|\frac{\varepsilon_a - \varepsilon_b}{\varepsilon_a + \varepsilon_b}\right|.$ (3)

It follows from here that in our problem $\varepsilon_2(\omega) = \varepsilon_1(\omega)$, $\beta_2 = \beta_1$, $R_2 = R_1$, $\alpha_2 = \alpha_1$. For considering the experimental values of the $Ti_3C_2T_x$ dielectric constant, we adapt the real and imaginary parts of the refractive index measured in the 450-550 nm range, $n \approx 2.7$ and $k \approx 1.0$[ii], respectively, yielding $\varepsilon_b \approx 6.3$. As for the dielectric functions of Ag and Au, we use the results of [[iii]]. The developed analytical approach provides good agreement with our UV-vis measurements, as presented in the Discussion section of the main text.

Calculation of the observed surface plasmon resonant (SPR) peak position in Pd@MXene

For the sheet-like planar particle on the surface of MXene flakes the condition of appearing of SPR can be found from the general expression for polarizability of oblate spheroid[iv]

$$\alpha(\omega) = \frac{\varepsilon(\omega) - \varepsilon_{eff}}{\varepsilon_{eff} + L\left(\varepsilon(\omega) - \varepsilon_{eff}\right)} V, \qquad (4)$$

where $\varepsilon(\omega)$ is the dielectric function of Pd, the dielectric constant of environment ε_{eff} contains the contribution of both media – water and MXene and is defined below, L is depolarization factor of spheroid and V is the volume of nanoparticle. We model sheet-like planar particle as disc i.e. strongly oblate spheroid (L=1) for which exp. (4) is reduced to $\alpha(\omega) = V\left(\varepsilon(\omega) - \varepsilon_{eff}\right)/\varepsilon(\omega)$ and consequently the condition for SPR is $\varepsilon(\omega) = 0$. According to the data of [see the reference in the main article, as

40] this condition is satisfied for $\lambda = 160\,\text{nm}$, which corresponds to UV region.

Interpretation of linewidth of observed resonances in Ag@MXene and Au@MXene: We also determine the shapes of the absorption spectra of Au and Ag spherical particles conditioned by SPR. It is known that the frequency dependent part $Q(\omega)$ of the absorption cross-section can be represented as follows

$$Q(\omega) = \omega \mathrm{Im}\alpha(\omega), \qquad (5)$$

where $\alpha(\omega)$ is the polarizability of the NP, depending on the dielectric function of the metal $\varepsilon(\omega)$ as well as on the dielectric properties of the surrounding, ε_{eff}, which contains the contribution of both media – water and MXene. This phenomenological approach (as will be shown below) allows calculating the radiation damping rate of Au and Ag NPs in a colloid with unknown dielectric properties steming from the concentration of MXenes, their size and functionalization.

In quasi-static approximation when the radius R of a NP is smaller than the resonance wavelength ($2\pi\omega \gg R$) we have[v]

$$\alpha(\omega) = \frac{\varepsilon(\omega) - \varepsilon_{eff}}{\varepsilon(\omega) + 2\varepsilon_{eff}} R^3. \qquad (6)$$

To use the well-known experimental data of refractive index, $n(\omega)$, and absorption coefficient, $k(\omega)$, for Ag and Au [iii], it is convenient to represent $Q(\omega)$ in the following form

$$Q(\omega) = \omega \mathrm{Im}\left(\frac{n^2(\omega) - k^2(\omega) + 2in(\omega)k(\omega) - n_{eff}^2}{n^2(\omega) - k^2(\omega) + 2in(\omega)k(\omega) + 2n_{eff}^2}\right) R^3, \qquad (7)$$

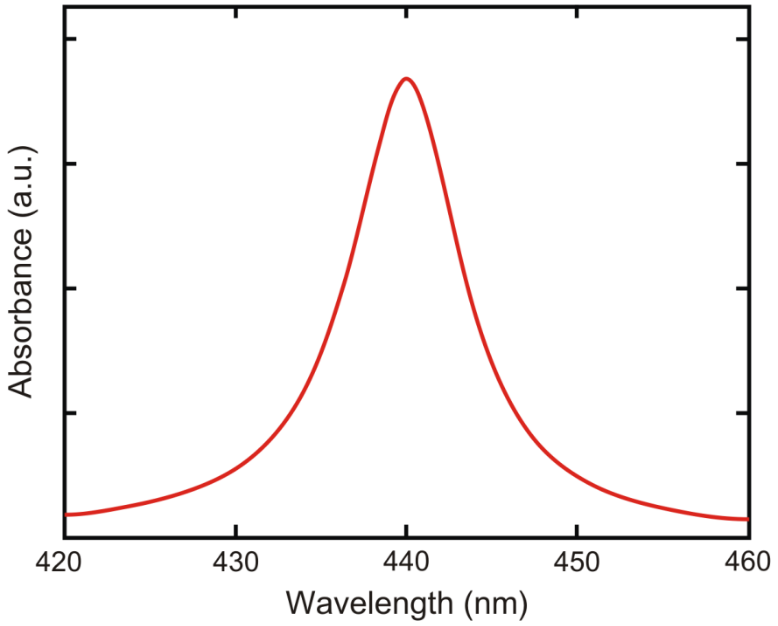

Figure [Sa]

where $n_{eff} = \sqrt{\varepsilon_{eff}}$. Here ε_{eff} is a fitting parameter, accounting for the joint effect of MXene and water on the absorption spectra of NPs. We perform the fitting procedure of ε_{eff} in a way so the calculated SPR frequencies match well with the experimental ones from Figure [2] for the same value of ε_{eff}, both for Ag and Au NPs. Thus, we calculate that a value of $n_{eff} = 1.805$ satisfies the above mentioned requirement. This rather high value of n_{eff} is conditioned mainly by the presence of MXene. The absorption spectra of Ag and Au NPs, respectively, calculated according to (7) with the use of the data of [iii] are presented in Figure [Sa] and [Sb].

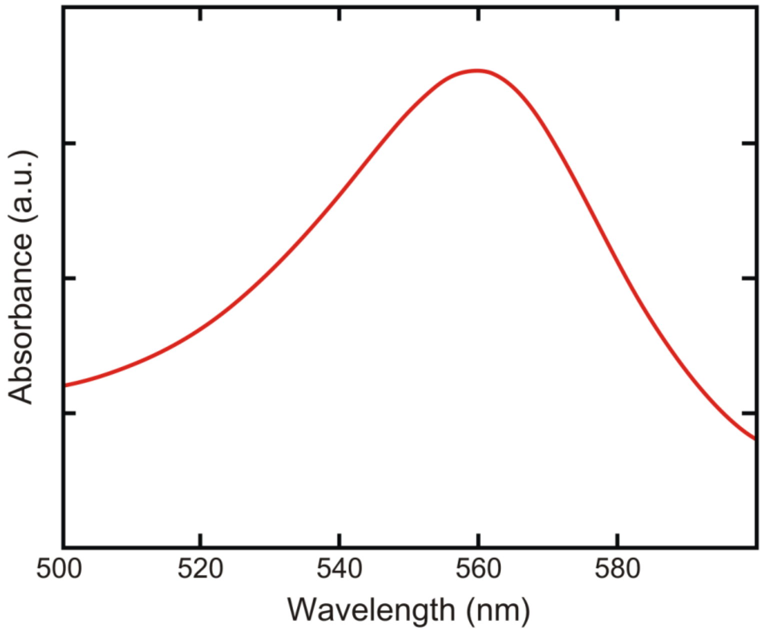

Figure [Sb]

Comparison of Figure [2] (experimentally obtained, refer main article) with Figure [Sa] and [Sb] shows excellent agreement with the observed SPR wavelength, λ_{SPR}, 440 nm for Ag and 558 nm for Au. Note that the full width at half maximum (FWHM) of the calculated SPR in Figures [Sa and Sb] are conditioned only by interband transitions and electron-phonon interaction, since they are plotted based on the optical constants of bulk Ag and Au [iii]. Obviously these data do not contain the contributions coming from the radiation damping of SPR oscillations since they do not appear in bulk samples. Thus to interpret the experimental data obtained for NPs adequately the contribution of radiation damping in the formation of the SPR linewidth should be analyzed. As it was shown[vi] the radiation damping rate Γ in a spherical NP is:

$$\Gamma = \frac{2}{3}\left(\frac{\omega_{SPR}R}{c}\right)^3 \omega_{SPR}\sqrt{\varepsilon_{eff}}\ , \quad (8)$$

where c is the speed of light in a vacuum. The dependence of Γ on the particle radius is rather strong, and when the dimensionless parameter $\omega_{SPR}R/c$ approaches unity,

i.e. Γ becomes comparable to ω_{SPR}, the radiative losses dominate.

Calculation of SPR radiative linewidth according to (8) for Ag spheres for the following measured values of parameters R = 35 nm, $\omega_{SPR} = 2.82$ eV (λ_{SPR}=440 nm), and determined above refractive index $n_{eff} = 1.805$, gives $\Gamma = 0.484$ eV. Converting this quantity to wavelengths we obtain for FWHM the value $\Delta\lambda_{calc}$=75.7 nm, which exceeds the FWHM in the Figure [Sa] (8 nm) by nearly an order of magnitude. Hence, interband transitions and electron-phonon interactions do not play a significant role in the SPR broadening of the Ag NPs. To further extract the pure contribution of SPR absorption in the Ag NPs from experimental data of the Figure [2] (main article), one should deduce the absorption data of the delaminated MXene from the absorption data of the Ag@MXene. This procedure leads to the following value of FWHM, $\Delta\lambda_{exp}$=80 nm, which is close to the calculated one, 75.7 nm. Note that we chose the largest NP since the absorbance according to (8) is proportional to R^3 and the shape of the resonance is formed mainly by them.

Applying the same procedure in case of Au NPs with the experimental value ω_{SPR}= 2.21 eV (λ_{SPR} =558 nm) (see Figure [2] in main article), we obtain for $\Delta\lambda_{exp}$=52 nm, which is close to the value extracted from the Fig. SB, $\Delta\lambda_{calc}$=60 nm. For the radiation damping rate, we obtain from expression (8) the value Γ =0.067 eV, which corresponds to $\Delta\lambda_{rad}$=17 nm. Thus we conclude that in the case of Au NPs the SPR linewidth broadening is mostly conditioned by interband transitions, and the contribution of the radiation damping is 30%.

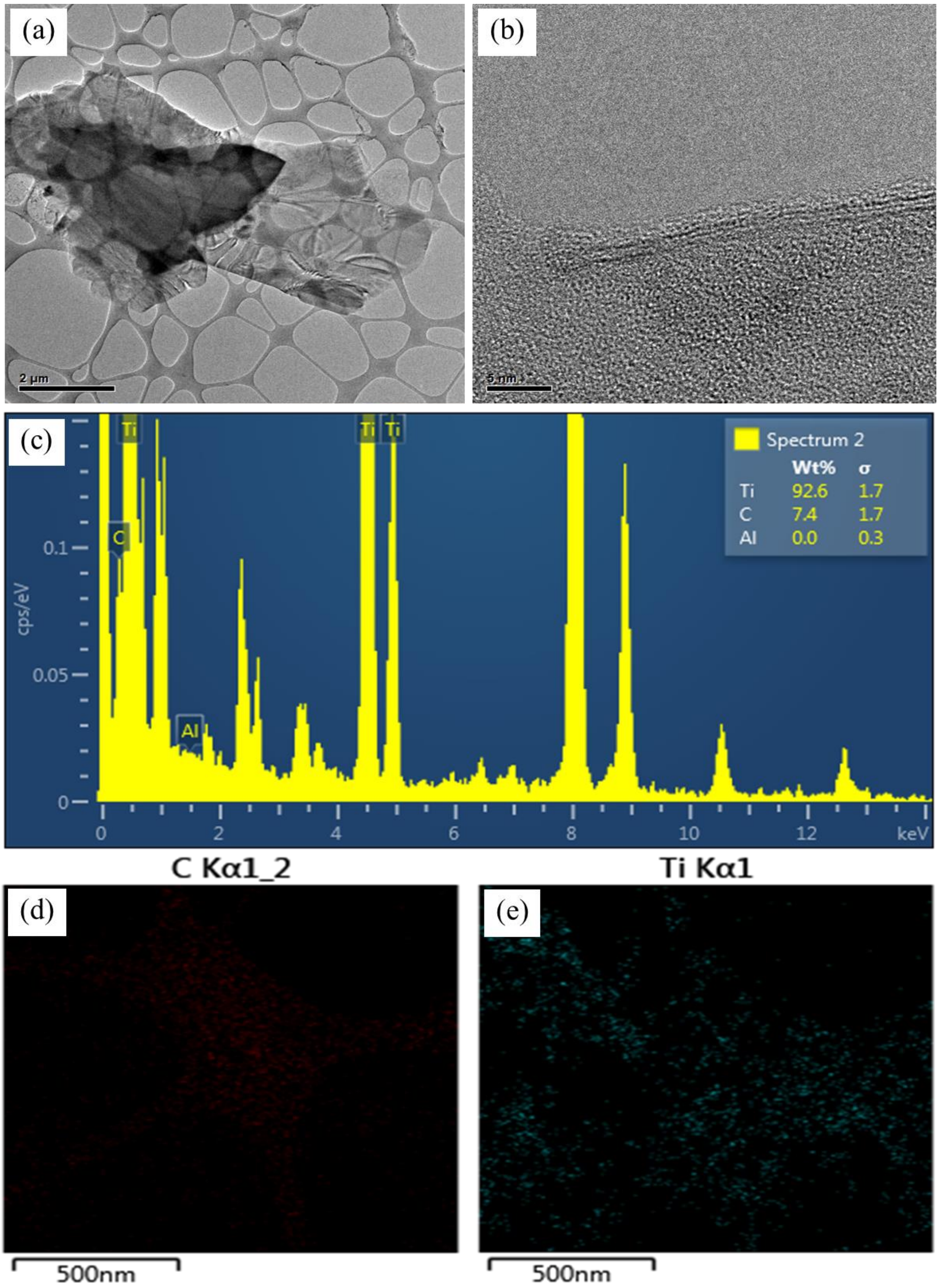

Figure [S1] Low magnification TEM images of delaminated MXene nanosheets (a), corresponding high-resolution TEM image of MXene (b), the EDX spectrum of delaminated MXene nanosheets (c) and their corresponding EDX elemental mapping analysis of Ti (d) and C (e).

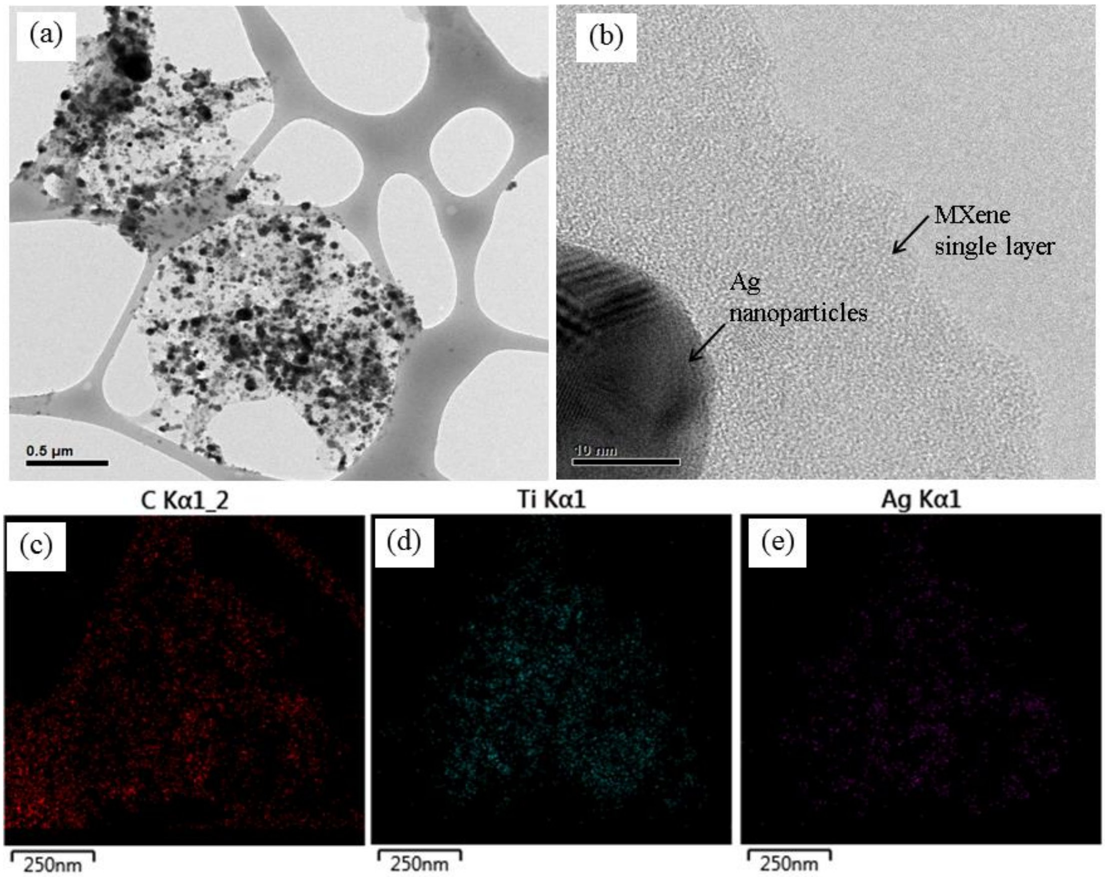

Figure [S2] Low magnification TEM image of Ag@MXene nanosheets (a), corresponding high-resolution TEM image of Ag@MXene (b), and their corresponding EDX elemental mapping analysis showing C (c), Ti (d) and Ag (e).

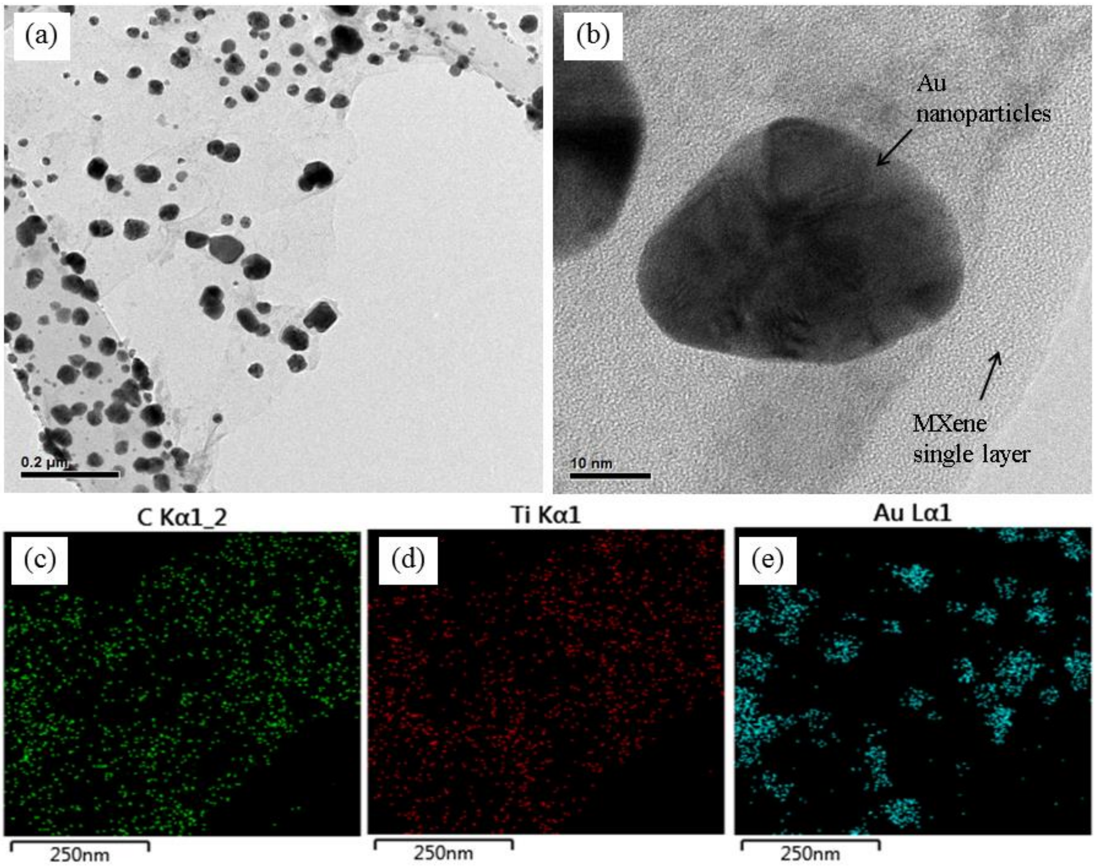

Figure [S3] Low magnification TEM image of Au@MXene nanosheets (a), corresponding high-resolution TEM image of Au@MXene (b), and their corresponding EDX elemental mapping analysis of C (c), Ti (d) and Au (e).

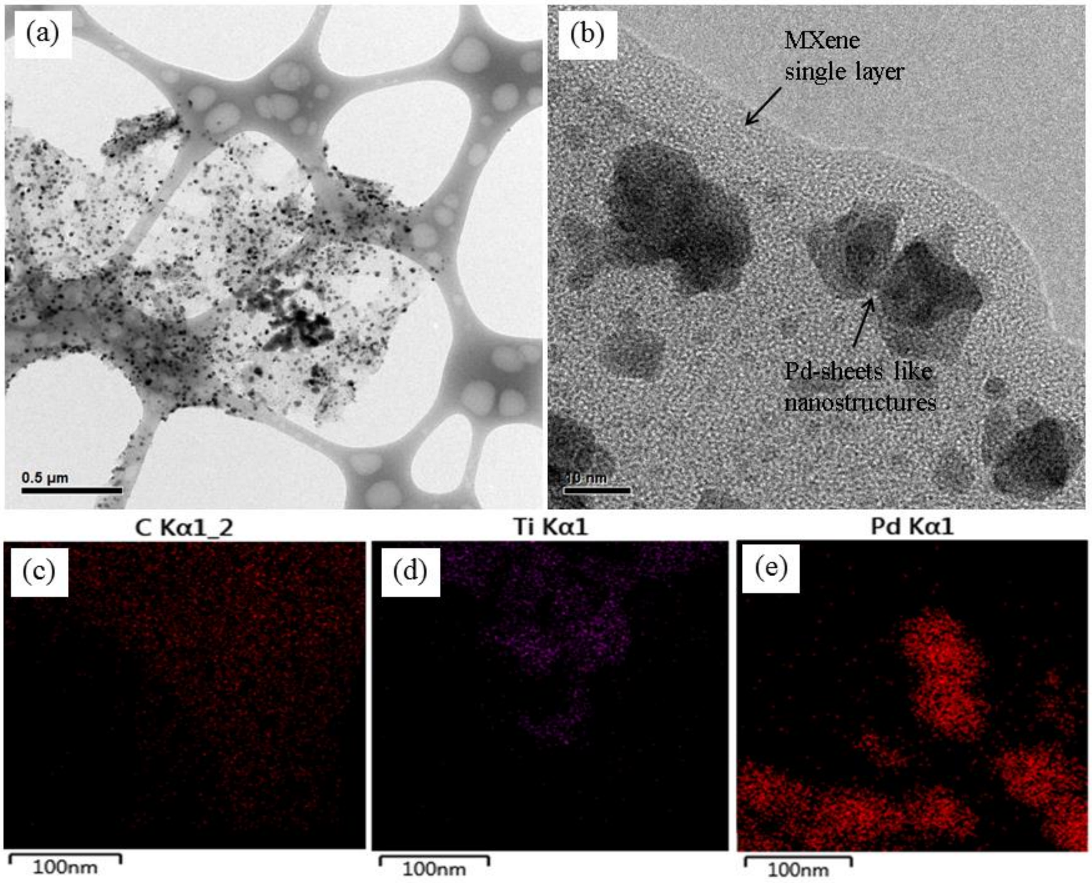

Figure [S4] Low magnification TEM image of Pd@MXene nanosheets (a), corresponding high-resolution TEM image of Pd@MXene (b), and their corresponding EDX elemental mapping analysis of C (c), Ti (d) and Pd (e).

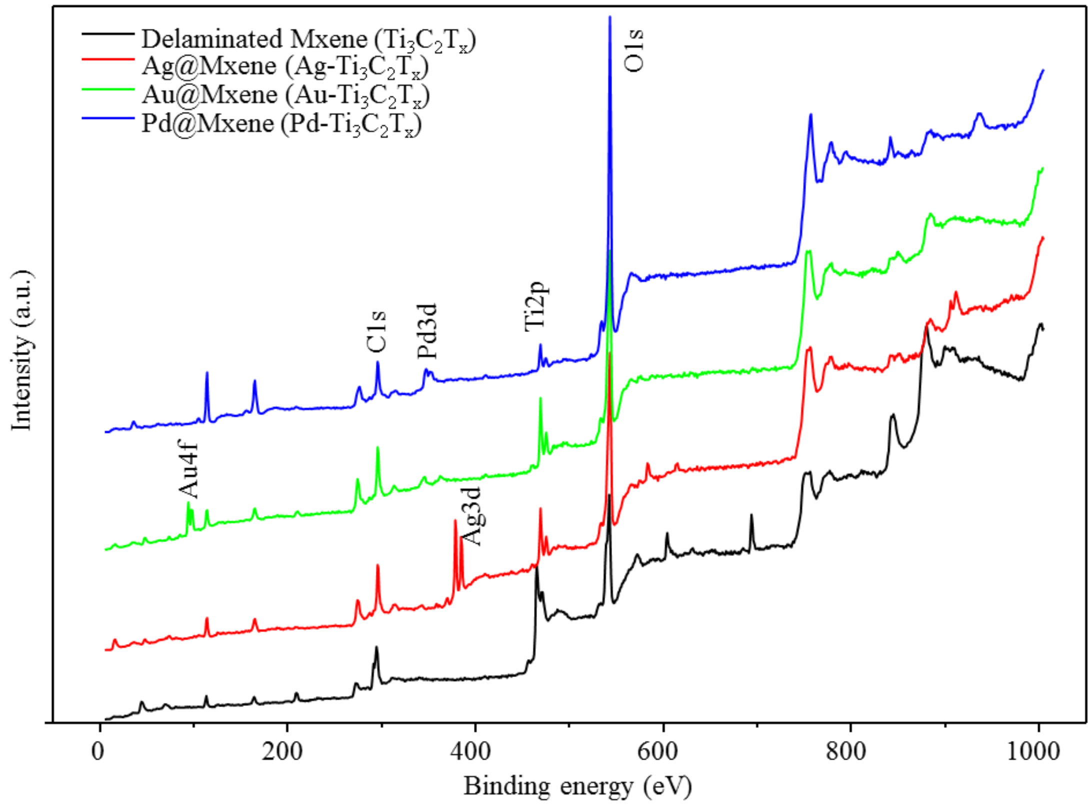

Figure [S5] A survey XPS spectrum of delaminated MXene nanosheets ($Ti_3C_2T_x$) and Ag@, Au@ and Pd@MXene hybrids.

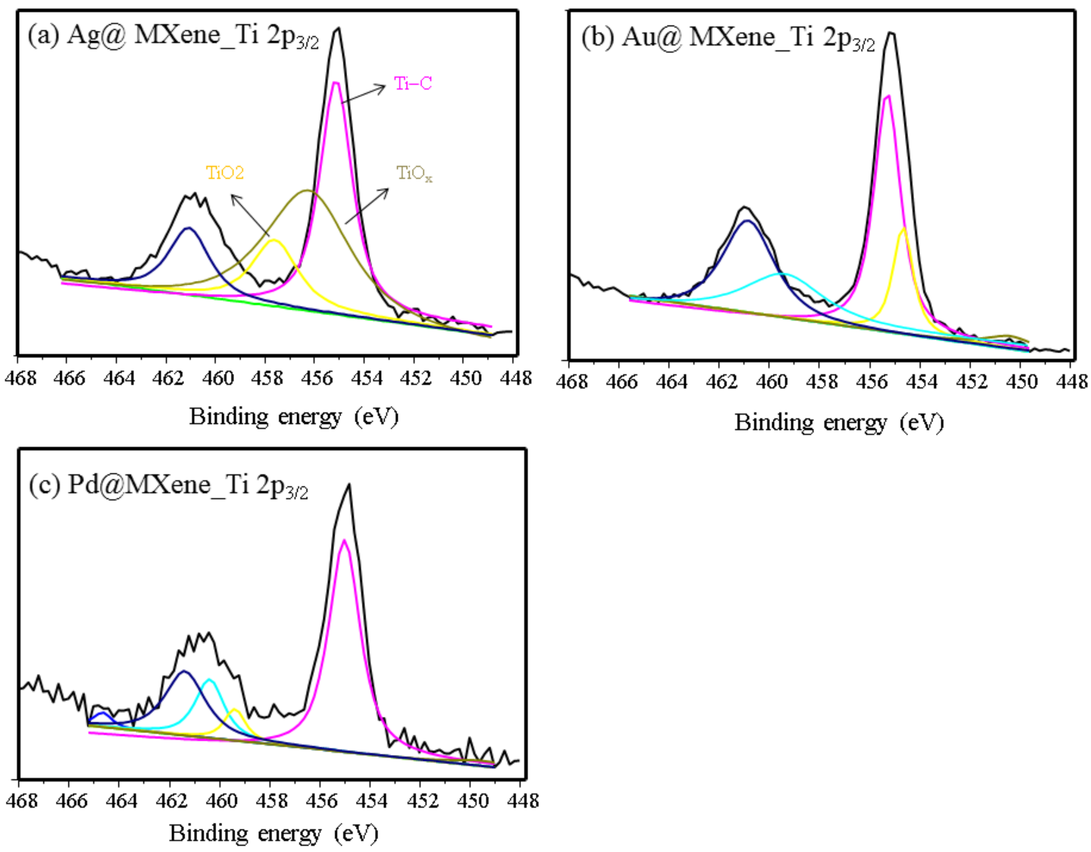

Figure [S6] High resolution XPS individual deconvolution spectrum of Ti $2p_{3/2}$ analysed (a) Ag@, (b) Au@ and (c) Pd@MXene hybrids. .

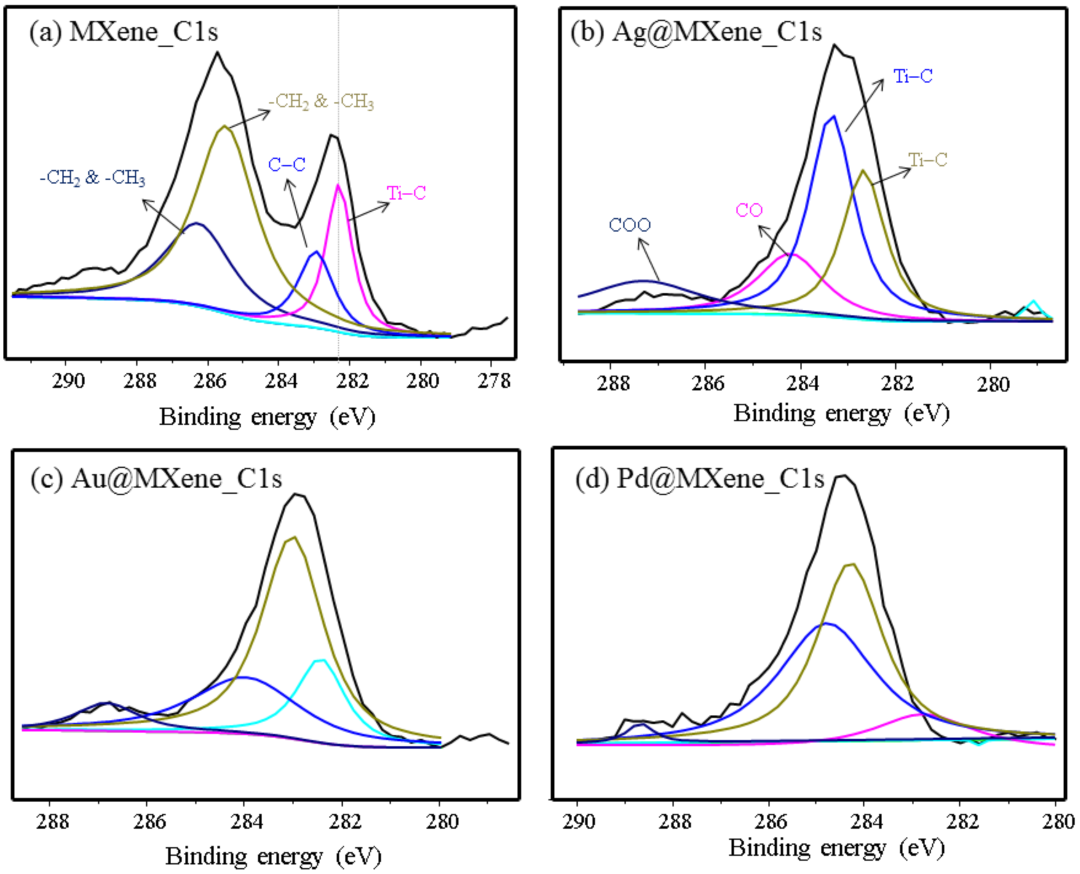

Figure [S7] High resolution XPS deconvolution spectrum of C 1s for for (a) MXene, and (b)Ag@, (c) Au@ and (d) Pd@MXene hybrids.

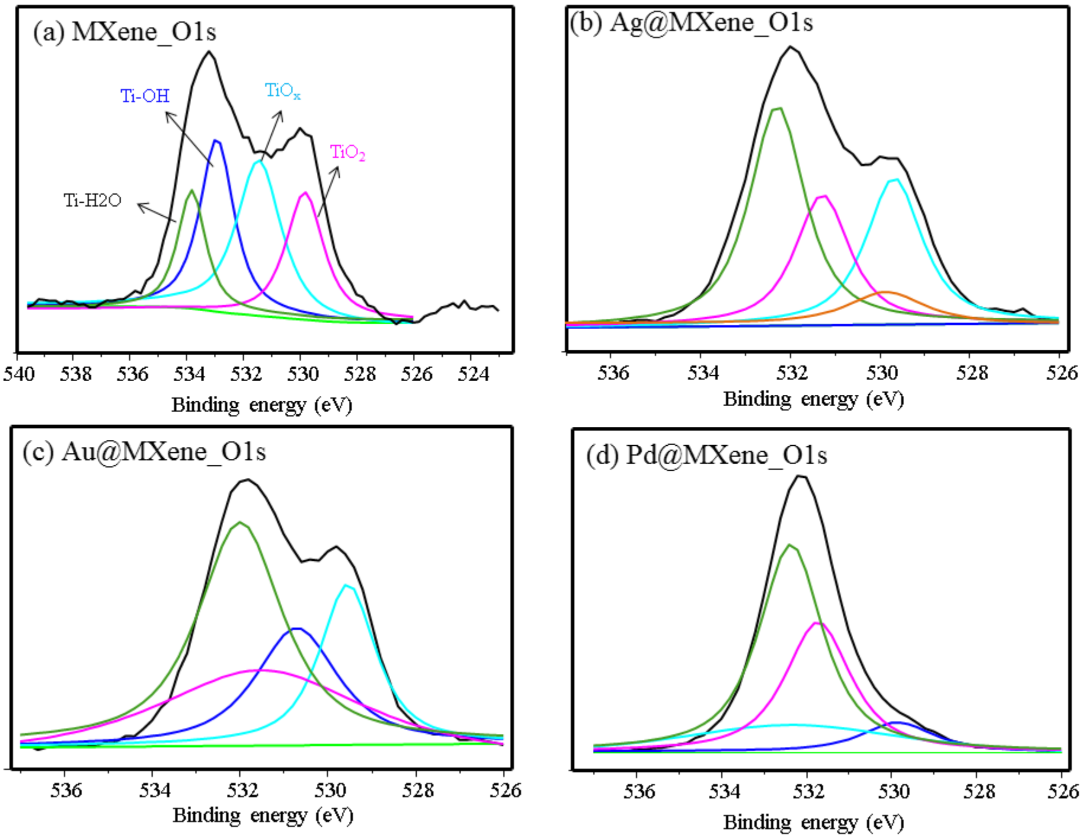

Figure [S8] High resolution XPS spectrum of O 1s for for (a) MXene, and (b) Ag@, (c) Au@ and (d) Pd@MXene hybrids.

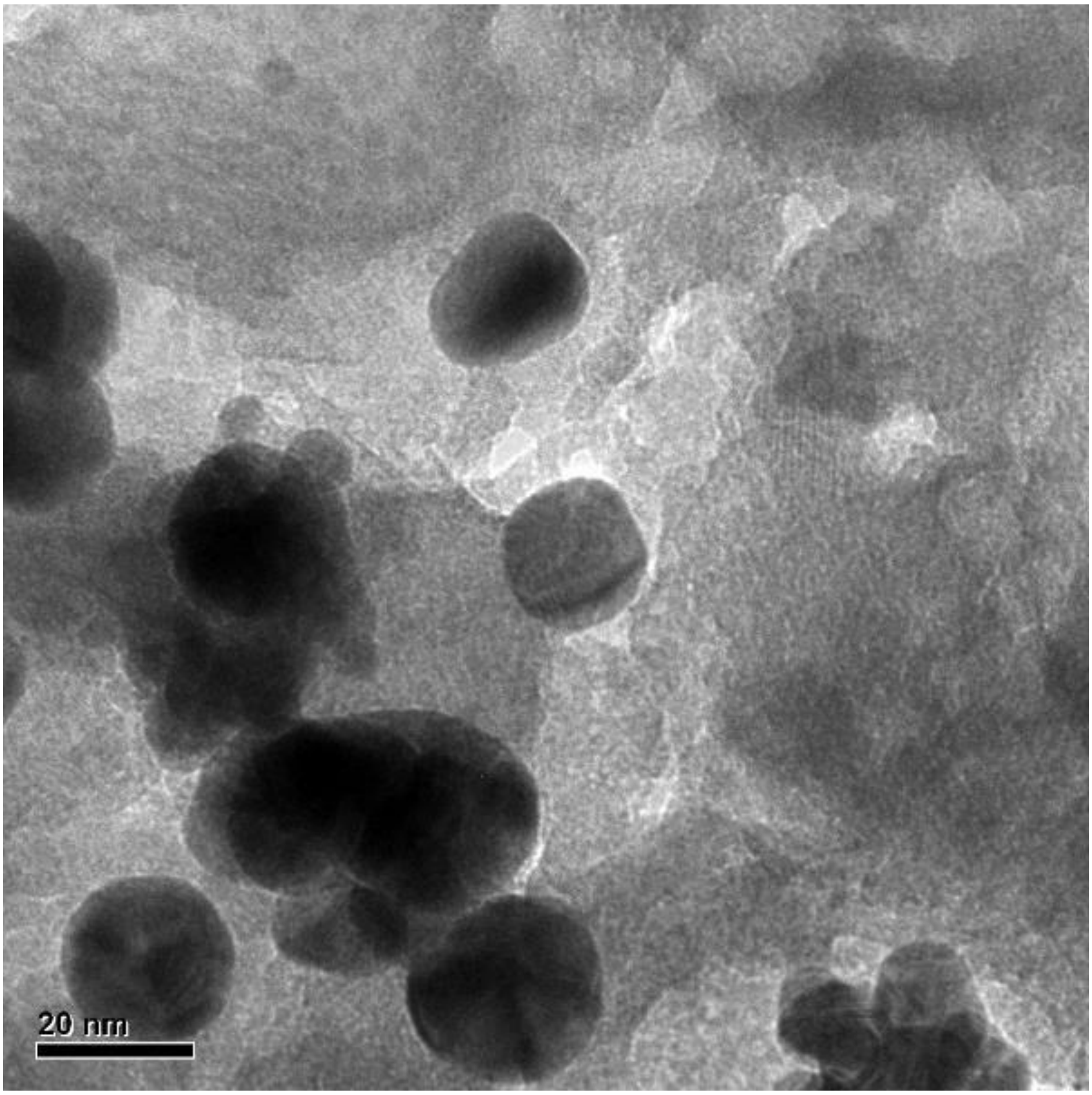

Figure [S9] High resolution TEM image of $Au@Ti_3C_2T_x$ prepared by without ultrasonication.

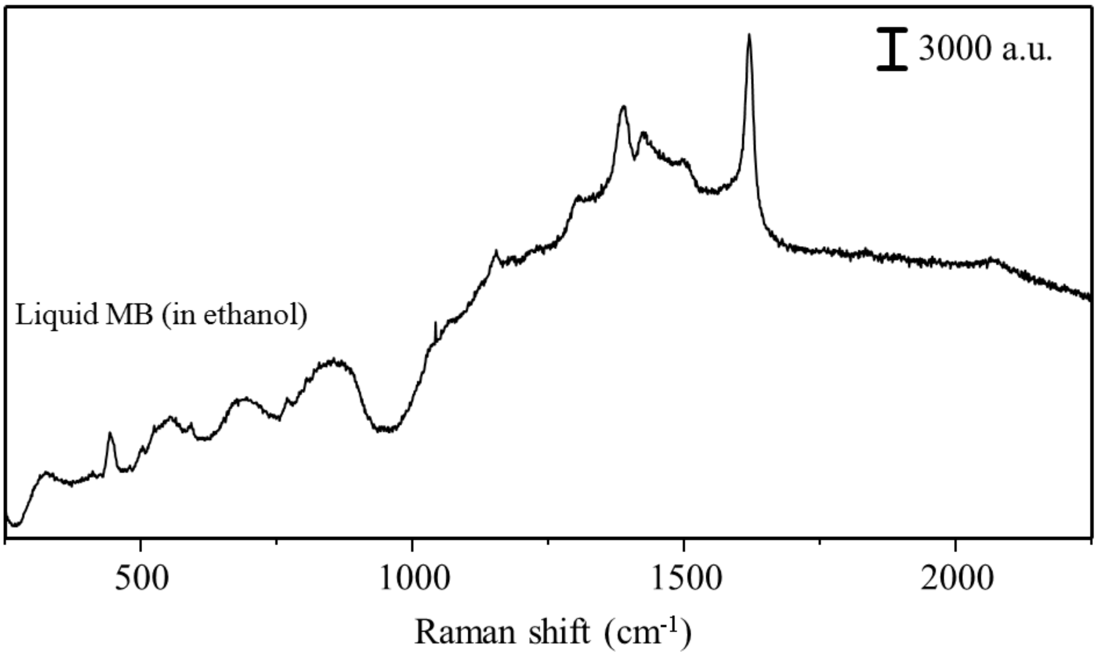

Figure [S10] The normal Raman spectra of methylene blue (MB) in ethanol solution.

Reference (Supplementary Information only).

[i]Chergui, M., Melikyan, A. & Minassian, H. Calculation of Surface Plasmon Frequencies of Two, Three and Four Strongly Interacting Nanospheres. *J. Phys. Chem. C,* **113**, 6463-6471 (2009).

[ii] Dillon, A. D., Ghidiu, M. J., Krick, A. L., Griggs, J., May, S. J., Gogotsi, Y., Barsoum, M. W. & Fafarman, A. T. Highly Conductive Optical Quality Solution-Processed Films of 2D Titanium Carbide. *Adv. Funct. Mater.* (2016) doi:10.1002/adfm.201600357

[iii] Johnson, P. B., Christy, R. W. *Phys Rev B,* ***6,*** 4370- 4379 (1972)

[v]A.Melikyan, H.Minassian, On Surface Plasmon Damping in Metallic Nanoparticles. *Applied Physics B*, Lasers and Optics, 78(3-4), pp. 455-457(2004).

[v] Kreibig, U. & Vollmer, M. Optical Properties of Metal Clusters; Berlin: Springer, 1995.

[vi] Melikyan, A. & H. Minassian. On Surface Plasmon Damping in Metallic Nanoparticles. *Applied Physics B*, Lasers and Optics, **78**, 455-457 (2004).
